# Uncovering the Role of Gut Microbiota in Amino Acid Metabolic Disturbances in Heart Failure Through Metagenomic Analysis

**DOI:** 10.3389/fcvm.2021.789325

**Published:** 2021-11-29

**Authors:** Tomohiro Hayashi, Tomoya Yamashita, Tomoya Takahashi, Tokiko Tabata, Hikaru Watanabe, Yasuhiro Gotoh, Masakazu Shinohara, Kenjiro Kami, Hidekazu Tanaka, Kensuke Matsumoto, Tetsuya Hayashi, Takuji Yamada, Ken-ichi Hirata

**Affiliations:** ^1^Division of Cardiovascular Medicine, Department of Internal Medicine, Kobe University Graduate School of Medicine, Kobe, Japan; ^2^Cardiovascular Division, Department of Medicine, Center for Cardiovascular Research, Washington University School of Medicine, St. Louis, MO, United States; ^3^School of Life Science and Technology, Tokyo Institute of Technology, Tokyo, Japan; ^4^Department of Bacteriology, Faculty of Medical Sciences, Kyushu University, Fukuoka, Japan; ^5^Division of Epidemiology, Kobe University Graduate School of Medicine, Kobe, Japan; ^6^The Integrated Center for Mass Spectrometry, Kobe University Graduate School of Medicine, Kobe, Japan; ^7^Human Metabolome Technologies, Tsuruoka, Japan

**Keywords:** heart failure, gut microbiota, amino acid (AA), metabolism, metagenomic analysis

## Abstract

**Aims:** Circulating amino acid (AA) abnormalities serve as predictors of adverse outcomes in patients with heart failure (HF). However, the role of the gut microbiota in AA disturbances remains unknown. Thus, we investigated gut microbial functions and their associations with AA metabolic alterations in patients with HF.

**Methods and Results:** We performed whole-genome shotgun sequencing of fecal samples and mass spectrometry-based profiling of AAs in patients with compensated HF. Plasma levels of total essential AAs (EAAs) and histidine were significantly lower in patients with HF than in control subjects. HF patients also displayed increased and decreased abundance of gut microbial genes involved in the degradation and biosynthesis, respectively, of EAAs, including branched-chain AAs (BCAAs) and histidine. Importantly, a significant positive correlation was observed between the abundance of microbial genes involved in BCAA biosynthesis and plasma BCAA levels in patients with HF, but not in controls. Moreover, network analysis revealed that the depletion of *Eubacterium* and *Prevotella*, which harbor genes for BCAA and histidine biosynthesis, contributed to decreased abundance of microbial genes involved in the biosynthesis of those EAAs in patients with HF.

**Conclusions:** The present study demonstrated the relationship between gut microbiota and AA metabolic disturbances in patients with HF.

## Introduction

Heart failure (HF) is currently recognized as a systemic disease that affects not only the heart, but also other organs such as the liver, kidney, and gut. Patients with chronic HF display alterations in morphology, permeability, and specific absorption in the intestine. These abnormalities are partially attributable to intestinal microcirculatory injuries (1–3). Malnutrition is an indicator of poor prognosis in patients with HF (4, 5). Multiple factors reduce blood amino acid (AA) levels in patients with HF, such as overconsumption in the heart and skeletal muscles, inadequate intake, and decreased absorption of nutrients (6).

The gut microbiota has the capacity to synthesize essential AAs (EAAs) *de novo* (7, 8). Importantly, a recent study has shown that gut microbial biosynthesis of branched-chain AAs (BCAAs; include leucine, isoleucine, and valine) was positively correlated with circulating levels of BCAAs in a healthy population (9). This suggests that AAs generated by the gut microbiota enter the bloodstream from the intestine and affect systemic AA levels in the host.

Previous studies have observed alterations in the compositions of gut microbiota using 16S ribosomal RNA (rRNA) gene sequencing in patients with HF (10, 11). While the human gut microbiota produces several metabolites that accumulate in the bloodstream and systemically influence the host (12–14), most research on HF has focused on the microbiota-dependent metabolite trimethylamine N-oxide (TMAO). High blood levels of TMAO were found to be associated with poor outcomes in patients with HF (15, 16) as well as cardiac dysfunction in experimental murine models (17, 18). Hence, gut microbial dysbiosis or microbe-generated metabolites are considered potential therapeutic targets for HF. However, the role of the gut microbiota in AA metabolic disturbances in HF remains unknown.

Here, we conducted whole metagenomic shotgun sequencing, allowing for the identification of the functional potential of the gut microbiome, and used a mass spectrometry-based approach to investigate whether alterations in the microbiota are involved in altered circulating AA levels in HF patients. Additionally, we measured fecal AA levels, which aid in investigating host-gut-interactions.

## Methods

### Study Population

Twenty-two HF patients were prospectively enrolled who were admitted for *de novo* acute decompensated HF or acute worsening of chronic HF. The diagnosis of HF was based on modified Framingham criteria (19, 20). We performed *post-hoc* whole-genome shotgun sequencing of fecal samples from 22 patients with HF as well as 11 age-, sex-, and comorbidity-matched control subjects admitted to Kobe University Hospital. In our previous work, the gut microbiota of the patients were determined using 16S rRNA sequencing and HF patients had a distinct gut microbial composition compared to control subjects (10). The control group had HF risk factors but no history of HF, among which 8 and 3 were classified as stage A and B, respectively. Control subjects comprised 2 patients who were admitted for the treatment of type 2 diabetes in the Division of Diabetes and Endocrinology and 9 patients who were admitted in the Division of Cardiovascular Medicine (6 patients for catheter ablation of atrial fibrillation, 2 patients for elective diagnostic coronary angiography, and 1 patient for symptomatic sick sinus syndrome requiring permanent pacemaker implantation). Both patients with HF and control subjects were maintained on hospital diets.

Blood and fecal samples from patients with HF were collected while in their compensated states during hospitalization. HF was considered as compensated if the following conditions were fulfilled: improvement of HF symptoms, amelioration of edema and pulmonary rales, withdrawal of intravenous diuretic and inotropic agents, and optimization of oral diuretics. Blood and fecal samples of controls were collected before catheterization or pacemaker implantation. The exclusion criteria of HF patients and control subjects included the following: acute coronary syndrome, renal failure (serum creatinine levels > 3.0 mg/dL at the time of admission), active infectious diseases, malignancy, autoimmune disorders, inflammatory or malabsorptive intestinal diseases, history of enterectomy, hepatic diseases such as hepatitis and liver cirrhosis, and use of antibiotics or steroid treatment within 1 month before admission and during hospitalization.

All study participants provided written informed consent prior to enrollment. The study was approved by the Ethics Committee of Kobe University (approval no. 160072 and 170131), conducted according to the principles outlined in the Declaration of Helsinki, and registered with the UMIN Clinical Trial Registry (trial registration no. UMIN000022414 and UMIN000015703).

### Measurement of Plasma AA Levels

Peripheral venous blood samples were collected in tubes containing EDTA-2Na and immediately centrifuged at 1,200 × *g* at 4°C for 10 min to obtain plasma, which were stored at −80°C for further analysis. The plasma samples were subjected to capillary electrophoresis time-of-flight mass spectrometry (CE-TOFMS) analysis to measure concentrations of ionic metabolites including AAs by Human Metabolome Technologies, as previously reported (10, 21).

### Measurement of Fecal AA Levels and DNA Extraction From Fecal Samples

The fecal samples collected from patients with HF and control subjects were stored at −80°C for further analysis, as described (10). The concentrations of AAs in the fecal samples were measured using liquid chromatography-tandem mass spectrometry (LC-MS/MS) with multiple reaction monitoring (MRM). We failed to measure AA level from the fecal sample of 1 control subject due to a shortage of the specimen.

For DNA extraction, 0.6 M perchloric acid and a stable isotope-labeled internal standard mixture (Wako, Osaka, Japan) were added to the fecal samples. Each mixture was then vigorously vortexed and shaken for 30 min at 4°C. After centrifugation, the resulting supernatants were passed through a 0.22 μm spin filter to remove particulates. The extracts were separated through liquid chromatography using an Intrada Amino Acid, 100 mm × 3 mm column (Imtakt Corporation, Kyoto, Japan). The DNA in the fecal samples were then extracted for whole-genome shotgun sequencing at Nihon Gene Research Laboratories, following an established procedure (22).

### Metagenome Sequencing of the Gut Microbiomes

The sequencing library was prepared using a QIAseq FX DNA Library Kit. Whole-genome shotgun sequencing of fecal samples were performed by Illumina Hiseq 2,500. All samples were paired-end sequenced with a 151-bp read length. Raw reads were performed on Platanus _trim version 1.0.7 (23) using default parameters to remove low-quality regions. To remove reads from the human genome, reads were mapped to GRCh38.p7 using BWA version 0.7.15 (24) with default parameters and subsequently removed using BLAT version 35 (25). Mapped reads with identity ≥ 60% and coverage ≥ 30% were removed. Finally, we obtained a total of 632,397,132 (15,809,928 on average) paired-end reads. To estimate the abundance of gene families, we analyzed the reads using HMP Unified Metabolic Analysis Network (HUMAnN2) version 0.11.1, as previously described (26, 27).

### Determining Alterations in Gut Microbial Compositions in Patients With HF

Linear discriminant analysis (LDA) effect size (LEfSe) (28) analysis was performed to determine the taxa contributing to the variations between the patients with HF and control subjects (logarithmic LDA score > 2.0 and alpha value <0.05).

### Kyoto Encyclopedia of Genes and Genomes (KEGG) Functional Analysis

Differentially enriched KEGG orthology (KO) modules were identified according to their reporter scores (29) from based on individual Z-scores. The KO was mapped to the KEGG module using the KEGG database (Release 97.0). The one-tailed Mann-Whitney U test was performed on all KOs occurring in more than five samples. The Z-scores of each KO were calculated as follows:


$$\begin{array}{l} {\text{Z}}_{\text{K}{\text{O}}_{\text{i}}}=\frac{{\text{P}}_{\text{K}{\text{O}}_{\text{i}}}-\mu }{\sigma }, \end{array}$$

Where *P*_*K*_*O*__*i*__ is the P-value of an individual KO, μ is the mean value of the KOs, and σ is the SD of KOs. The aggregated Z-score of the KEGG module was calculated as follows:


$$\begin{array}{l} {\text{Z}}_{\text{module}}=\;{\frac{1}{\sqrt{\text{k}}}}_{\text{K}{\text{O}}_{\text{i}}}, \end{array}$$

where k is the number of KOs in the module. We corrected the background distribution of the *Z*_module_ by collecting the aggregated Z-scores of 1,000 randomly chosen sets of k values KO and applying the following equation:


$$\begin{array}{l} {\text{Z}}_{\text{adjustedmodule}}=\frac{{\text{Z}}_{\text{module}}-\;\mu_{\text{k}}}{\sigma_{\text{k}}}, \end{array}$$

where μ_k_ is the mean value and σ_k_ is the SD. The Z_adjustedmodule_ was then used as the final reporter score to evaluate the enrichment of specific modules. An absolute value of a reporter score of ≥1.6 (90% confidence according to normal distribution) was used to determine significantly different pathways (30). We used Spearman's correlation to show the associations of BCAAs and histidine in plasma and feces with the genes that synthesize, degrade, and transport them. Heatmaps of abundances and Spearman correlations were constructed using heatmap.2 from the gplots package in R software.

### Network Analysis

To visualize the enrichment of KOs in the module and their associated genera, a network diagram was drawn manually using yED version 3.1.8.11.

### Statistical Analysis

Bioinformatics analyses of the gut microbiota were performed as described above. Comparisons of plasma and fecal AA levels between patients with HF and control subjects were performed using Student's *t*-test or Mann-Whitney U test for normally and non-normally distributed data, respectively. Data are presented as mean ± SD or SEM for normally distributed data and as the median (25–75th percentiles) for non-normally distributed data. Categorical variables were compared between groups using Fisher's exact test. We examined the association between the two parameters using Spearman's correlation test. Data were analyzed using R software version 3.1.0 (http://www.r-project.org/), Prism version 7.0 (GraphPad Inc., San Diego, CA, USA), and JMP version 10 (SAS Institute, Cary, NC, USA). Statistical significance was set at *P* < 0.05.

## Results

### Alterations in the Gut Microbial Compositions in Patients With HF

**Figure 1:**
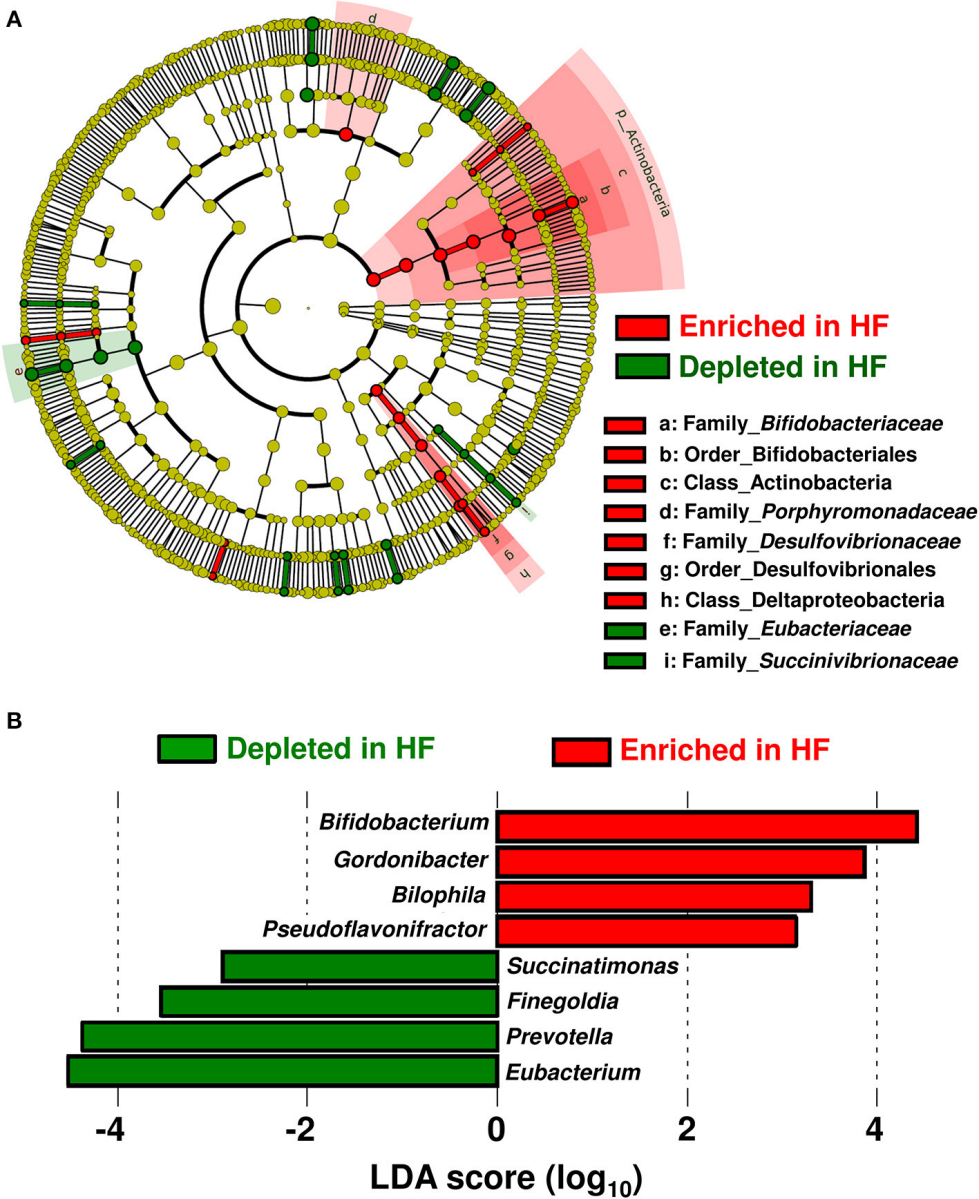
Compositional differences in gut microbiota between heart failure (HF) patients (*n* = 22) and controls (*n* = 11). **(A)** A cladogram demonstrating differential taxa at the class, order, and family levels between patients with HF and controls. Only the taxa with *P* < 0.05 and a linear discriminant analysis (LDA) significant threshold of > 2.0 are shown. **(B)** Taxa at the genus level. The enriched taxa in patients with HF and controls are presented as positive (red) and negative (green) LDA scores, respectively.

Table 1 shows the patients' characteristics. Patients with HF had higher circulating B-type natriuretic peptide (BNP) and high-sensitivity C-reactive protein levels but lower plasma albumin levels compared with control subjects. Beta blocker, aldosterone receptor antagonist, and diuretics (loop or thiazide) were used more in HF patients than in controls. The taxa enriched in patients with HF consisted of the Acinobacteria and Deltaproteobacteria classes; Bifidobacteriales and Desulfovibrionales orders; as well as *Bifidobacteriaceae, Porphyromonadaceae*, and *Desulfovibrionaceae* families. On the other hand, the *Eubacteriaceae* and *Succinivibrionaceae* families were depleted in patients with HF (Figure 1A).

**Table 1:** Patient characteristics.

	**Control subjects**	**HF patients**	***P*-value**
	**(*n* = 11)**	**(*n* = 22)**	
Age, years	72 ± 7	72 ± 18	0.955
Male sex	6 (55%)	14 (64%)	0.614
NYHA class I		3 (14%)	
NYHA class II		15 (68%)	
NYHA class III		4 (18%)	
NYHA class IV		0 (0%)	
Body mass index, kg/m^2^	24.4 ± 3.1	23.6 ± 5.9	0.701
Smoking	3 (27%)	11 (50%)	0.278
**Ejection fraction, %**			
≤ 40	0 (0%)	12 (55%)	
41–49	0 (0%)	0 (0%)	
≥50	11 (100%)	10 (45%)	
**Primary cause of HF**			
Ischemic heart disease		2 (9%)	
Dilated cardiomyopathy		7 (32%)	
Hypertension		4 (18%)	
Valvular disease		5 (23%)	
Others		4 (18%)	
**Comorbidities**			
Hypertension	9 (82%)	21 (95%)	0.252
Diabetes mellitus	5 (45%)	8 (36%)	0.614
Dyslipidemia	6 (55%)	9 (41%)	0.458
Atrial fibrillation	6 (55%)	15 (68%)	0.443
**Medication**			
ACEi and/or ARB	5 (45%)	15 (68%)	0.208
Beta blocker	4 (36%)	20 (91%)	0.002
Calcium channel blocker	3 (27%)	10 (45%)	0.456
Aldosterone receptor antagonist	0 (0%)	9 (41%)	0.015
Diuretics (loop or thiazide)	2 (18%)	21 (95%)	<0.001
Statin	5 (45%)	4 (18%)	0.121
PPI/H_2_ blocker	9 (82%)	17 (77%)	>0.999
Antidiabetic agent (including insulin therapy)	4 (36%)	5 (23%)	0.438
Antiplatelet	2 (18%)	5 (23%)	>0.999
Anticoagulant agent	6 (55%)	18 (82%)	0.121
Antibiotics	0 (0%)	0 (0%)	>0.999
Probiotics	0 (0%)	0 (0%)	>0.999
Immunosuppressive agent	0 (0%)	0 (0%)	>0.999
**Biochemical measures at time of admission**			
Albumin, g/dL	3.7 ± 0.3	3.2 ± 0.5	0.009
Hemoglobin, g/dL	13.3 ± 1.4	12.1 ± 2.5	0.155
Total bilirubin, mg/dL	1.0 ± 0.3	0.8 ± 0.3	0.077
AST, U/L	23.6 ± 6.0	25.4 ± 9.6	0.591
ALT, U/L	18.1 ± 5.1	21.1 ± 12.0	0.437
BUN, md/dL	20.0 (19.3–23.6)	21.4 (16.3–34.3)	0.313
Creatinine, mg/dL	1.0 ± 0.3	1.2 ± 0.5	0.109
eGFR, mL/min/1.73m^2^	54.0 ± 11.7	49.0 ± 22.5	0.496
hs-CRP, mg/dL	0.04 (0.03–0.06)	0.46 (0.06–1.51)	0.001
BNP, pg/mL	53 (23–109)	284 (152–378)	<0.001

*Binary data are presented as number (%); continuous variables are presented as mean (SD) or median (interquartile range). ACEi, angiotensin-converting enzyme inhibitor; ALT, alanine aminotransferase; ARB, angiotensin II receptor blocker; AST, aspartate aminotransferase; BNP, B-type natriuretic peptide; BUN, blood urea nitrogen; eGFR, estimated glomerular filtration rate; HF, heart failure; hs-CRP, high-sensitivity C-reactive protein; NYHA, New York Heart Association; PPI, proton pump inhibitor*.

At the genus level, eight genera were differentially enriched between patients with HF and control subjects. Genera such as *Bifidobacterium, Gordonibacter, Bilophila*, and *Pseudoflavonifractor* were abundant, whereas genera such as *Eubacterium, Prevotella, Finegoldia*, and *Succinatimonas* were significantly decreased in patients with HF (Figure 1B). The relative abundances and principal coordinate analysis based on genus-level gut microbiomes are presented in Supplementary Figure 1. A subgroup analysis based on BNP levels demonstrated that the severity of HF did not influence gut microbial compositions in HF patients (Supplementary Figure 2A). Gut microbial compositions were similar between HF patients with reduced ejection fraction (HFrEF) and preserved ejection fraction (HFpEF) (Supplementary Figure 2B). Whereas, the relative abundance of the genus-level gut microbiomes were affected by medications used to treat HF (Supplementary Figure 3).

### Plasma and Fecal AA Concentrations

**Figure 2:**
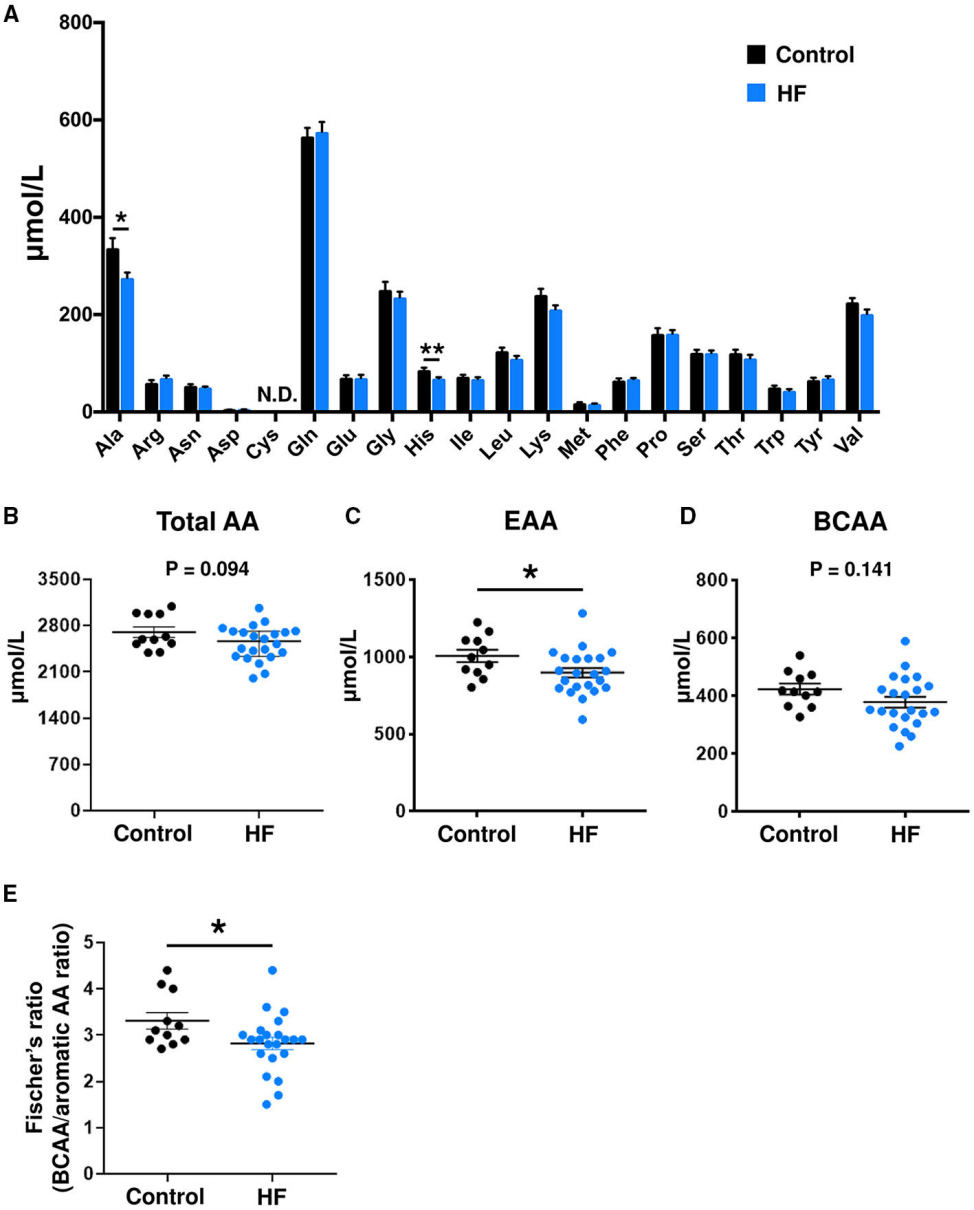
Comparison of plasma amino acid (AA) concentrations between heart failure (HF) patients (n = 22) and control subjects (*n* = 11). **(A)** Plasma concentrations of each AA. **(B)** Plasma concentrations of total AAs. **(C)** Plasma concentrations of total essential AAs (EAAs), including His, Ile, Leu, Lys, Met, Phe, Thr, Trp, and Val. **(D)** Plasma concentrations of branched chain AAs (BCAAs), including Leu, Ile, and Val. **(E)** Fischer's ratio, which is the ratio of BCAAs to aromatic AAs (Phe and Tyr). The data are presented as mean ± SEM. *P < 0.05, **P < 0.01. Ala, alanine; Arg, arginine; Asn, asparagine; Asp, aspartic acid; Cys, cysteine; Gln, glutamine; Glu, glutamic acid; Gly, glycine; His, histidine; Ile, isoleucine; Leu, leucine; Lys, lysine; Met, methionine; Phe, phenylalanine; Pro, proline; Ser, serine; Thr, threonine; Trp, tryptophan; Tyr, tyrosine; Val, valine; N.D., not detected.

Plasma and fecal AA concentrations were measured using mass spectrometry. Compared to controls, patients with HF demonstrated distinct metabolic plasma profiles (Supplementary Figure 4). The concentrations of 2 plasma AAs [alanine (*P* < 0.05) and histidine (*P* < 0.01)] were significantly lower in patients with HF (Figure 2A). Furthermore, the HF group had statistically significantly lower total plasma EAA levels than controls (*P* < 0.05) (Figure 2C). Patients with HF demonstrated decreases in concentrations of total plasma AAs (*P* = 0.094) and BCAAs (*P* = 0.141), but the differences were not statistically significant (Figures 2B,D). The Fischer's ratios (ratio of BCAA to aromatic AAs such as tyrosine and phenylalanine) of patients with HF were also lower than those in controls (*P* < 0.05) (Figure 2E). A significant inverse correlation was observed between circulating BNP and EAA levels (*r* = −0.492, *P* = 0.020) (Supplementary Figure 5A). There was no difference in plasma EAA levels between HFrEF and HFpEF patients (Supplementary Figure 5B). Plasma EAA levels did not differ between patients with or without medications for treating HF (Supplementary Figure 6).

**Figure 3:**
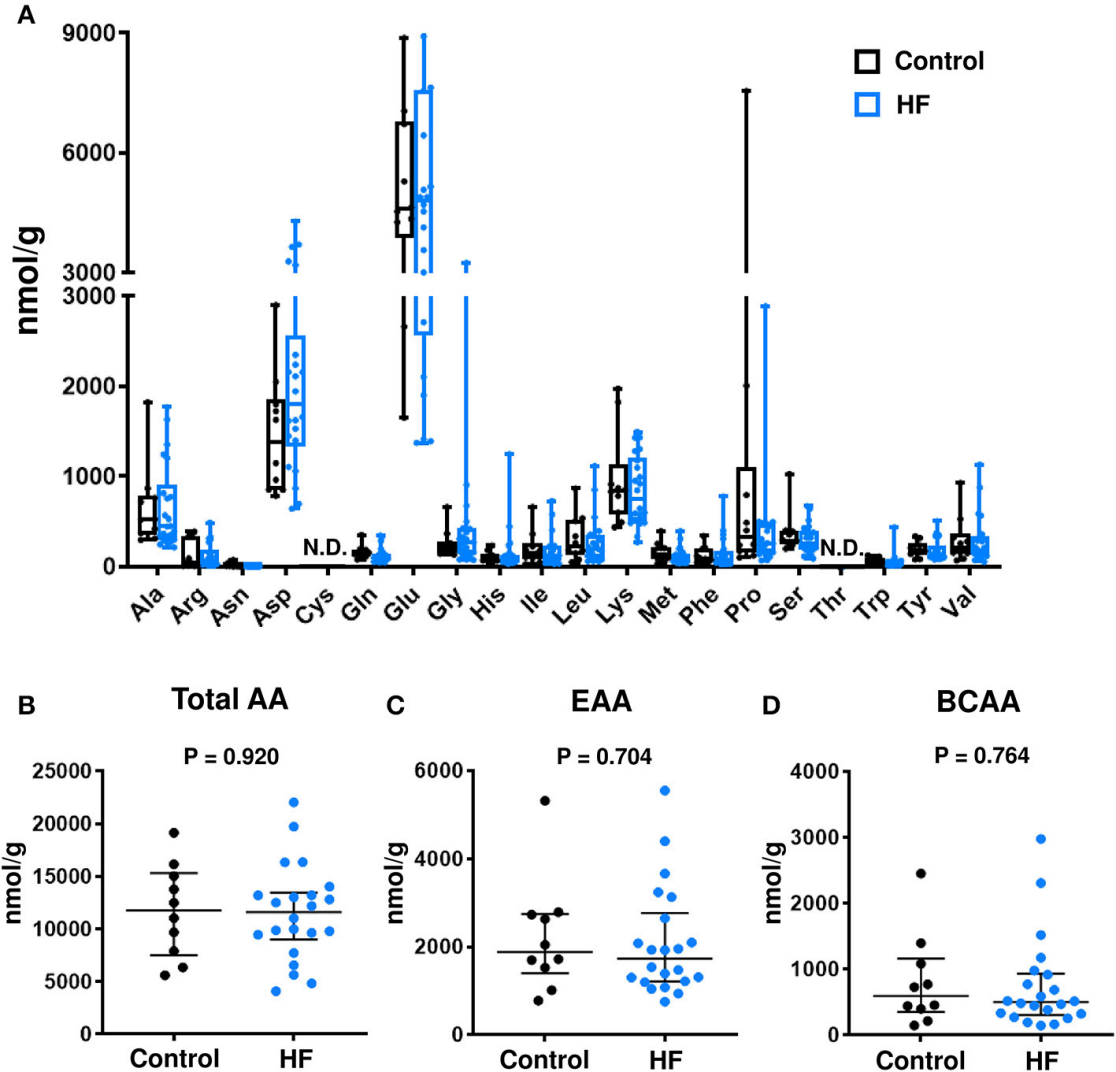
Comparison of fecal amino acid (AA) concentrations between heart failure (HF) patients (*n* = 22) and control subjects (*n* = 10). **(A)** Fecal concentrations of each AA. In the box-and-whisker plot, the middle line represents the median value, the box indicates interquartile range (25–75th percentiles), and the range bars indicate the maximum and minimum values. **(B)** Fecal concentrations of total AAs. **(C)** Fecal concentrations of total essential AAs (EAAs), including His, Ile, Leu, Lys, Met, Phe, Thr, Trp, and Val. **(D)** Fecal concentrations of branched chain AAs (BCAAs), including Leu, Ile, and Val. **(B–D)** Data are presented as median ± interquartile range (25–75th percentiles). Ala, alanine; Arg, arginine; Asn, asparagine; Asp, aspartic acid; Cys, cysteine; Gln, glutamine; Glu, glutamic acid; Gly, glycine; His, histidine; Ile, isoleucine; Leu, leucine; Lys, lysine; Met, methionine; Phe, phenylalanine; Pro, proline; Ser, serine; Thr, threonine; Trp, tryptophan; Tyr, tyrosine; Val, valine; N.D., not detected.

The concentrations of individual AAs (Figure 3A), total AAs (Figure 3B), total EAAs (Figure 3C), and total BCAAs (Figure 3D) were similar between the fecal samples of patients with HF and control subjects.

### Gut Microbial Functions and Their Correlations With Host AA Metabolism

**Figure 4:**
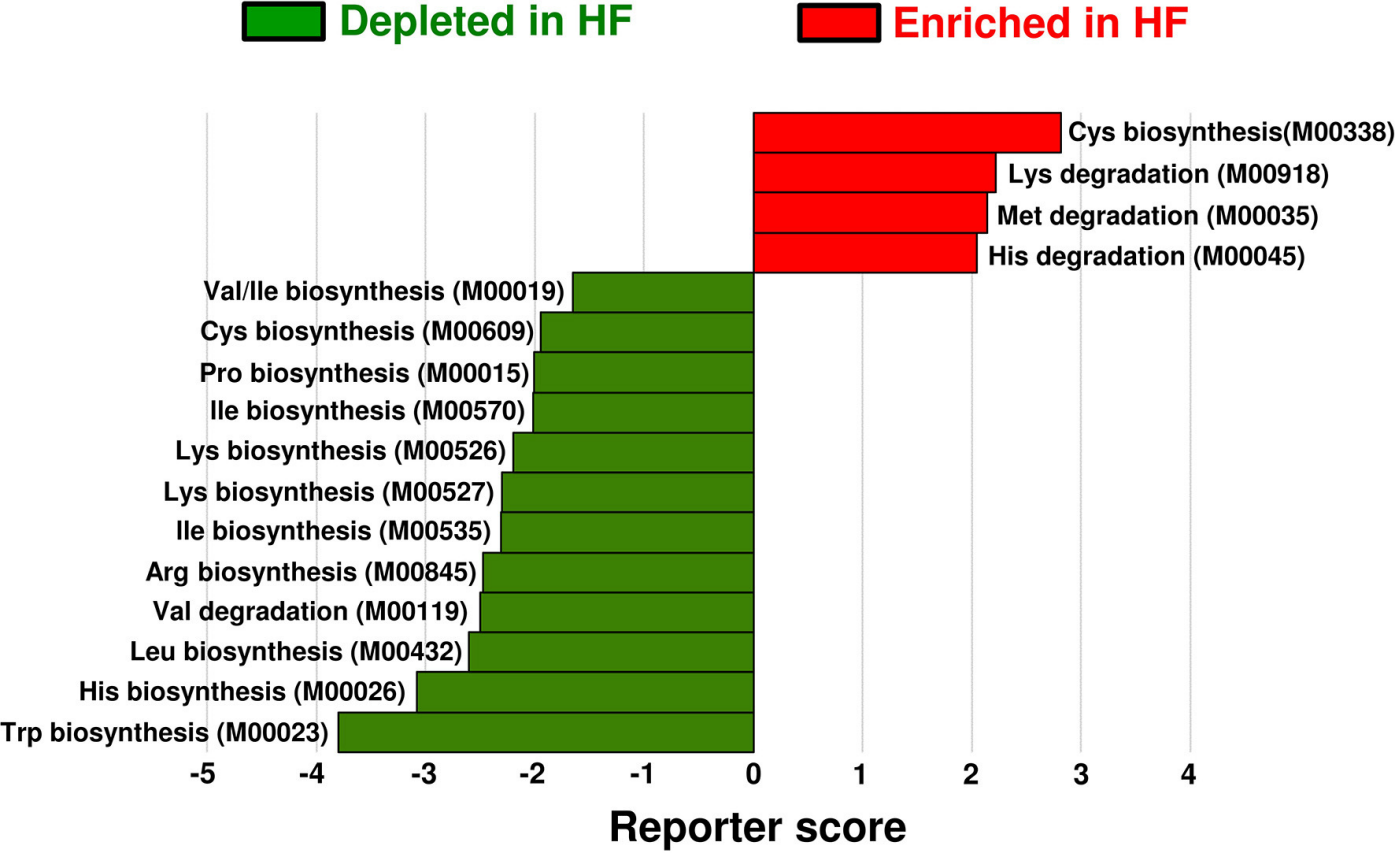
Functional alterations in gut microbial amino acid metabolism among patients with heart failure (HF). Differentially enriched Kyoto Encyclopedia of Genes and Genomes (KEGG) orthology modules in amino acid metabolism between patients with HF (*n* = 22) and controls (*n* = 11) are shown. The enriched modules in patients with HF and controls are indicated with positive reporter scores (red) and negative reporter scores (green), respectively. Arg, arginine; Cys, cysteine; His, histidine; Ile, isoleucine; Leu, leucine; Lys, lysine; Met, methionine; Pro, proline; Trp, tryptophan; Val, valine.

**Figure 5:**
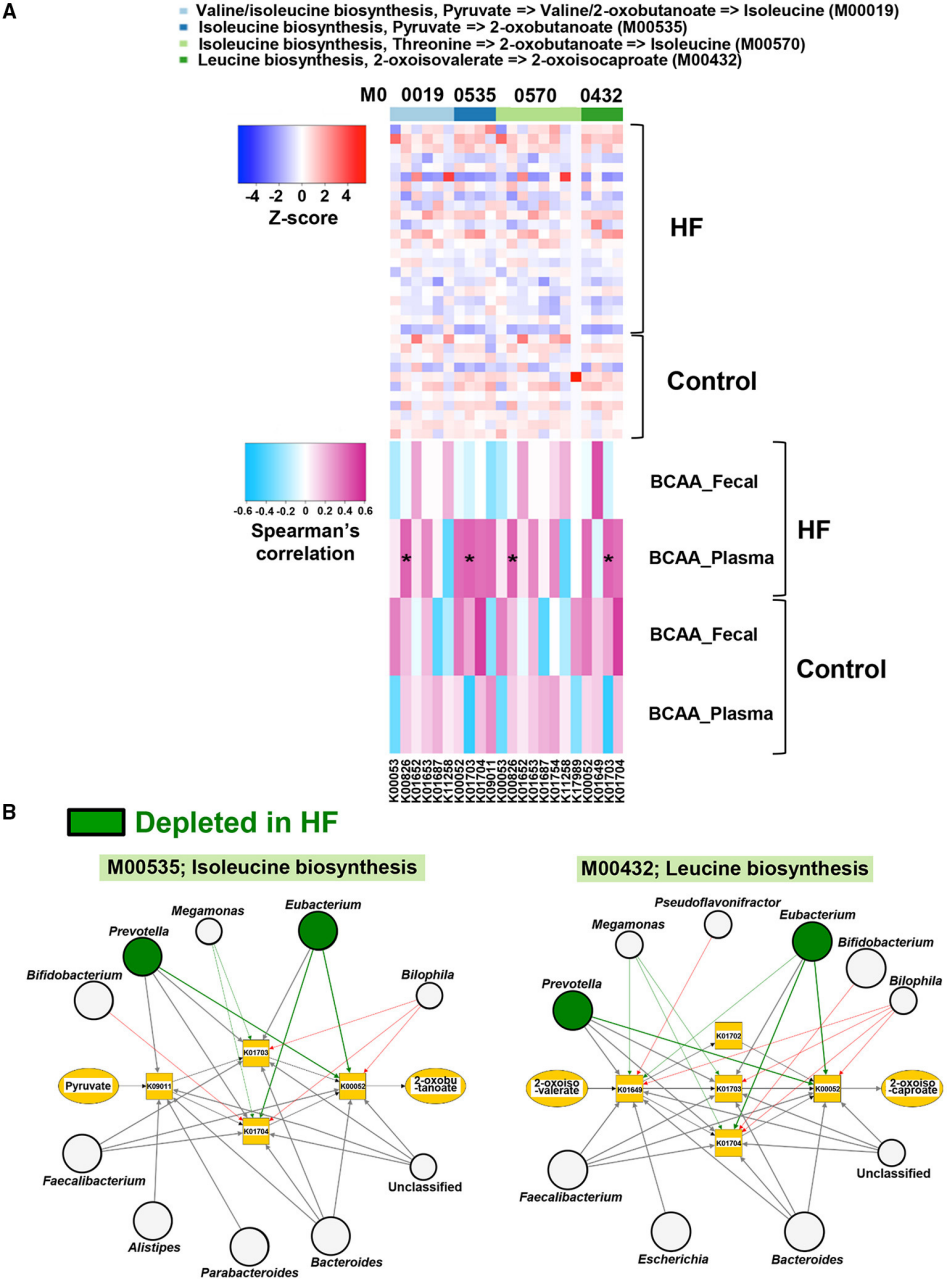
Correlations of gut microbial functions with host plasma and fecal branched chain amino acid (BCAA) concentrations along with the visualization of genera associated with BCAA biosynthesis. **(A)** Correlations of abundances of genes involved in BCAA biosynthesis with plasma and fecal BCAA concentrations in heart failure (HF) patients (*n* = 22) and controls (*n* = 11). **(B)** Kyoto Encyclopedia of Genes and Genomes (KEGG) orthology (KO) genes and their associated genera in BCAA biosynthesis (M00535 and M00432) are shown. The genera depleted in patients with HF (*n* = 22) are indicated by green circles. Arrows are drawn from the top 5 abundant genera in each KO gene (thick arrows) or from the genera with differentially enriched KO genes between patients with HF (*n* = 22) and controls (*n* = 11). Red arrows indicate that the KO genes possessed by each genus were significantly increased in patients with HF (*n* = 22). Green arrows indicate the KO genes significantly decreased in patients with HF (*n* = 22). Large circles indicate the top 10 abundant genera (i.e., *Bacteroides, Prevotella, Subdoligranulum, Eubacterium, Escherichia, Streptococcus, Bifidobacterium, Faecalibacterium, Alistipes*, and *Parabacteroides*) in all samples (*n* = 33). *P < 0.05.

To investigate the alterations in gut microbial functions between patients with HF and controls, we performed a KEGG functional analysis (Supplementary Table). The decreased abundance of microbial genes involved in the biosynthesis of several AAs, including BCAAs and histidine, was observed in patients with HF. Meanwhile, genes involved in the degradation of lysine, methionine, and histidine were increased in patients with HF (Figure 4). Intriguingly, correlation analysis revealed that the abundance of KEGG orthology (KO) genes related to the biosynthesis of BCAAs demonstrated significant positive correlation with plasma BCAA levels in patients with HF (Figure 5A, Supplementary Figure 7). Moreover, a modest positive correlation was observed between the abundance of microbial KO genes involved in the biosynthesis of histidine and the plasma histidine levels in patients with HF (Supplementary Figure 8). We also found that microbial genes involved in the degradation and transport of histidine demonstrated a modest inverse correlation with plasma histidine levels in patients with HF (Supplementary Figure 8).

### Specific Gut Microbes Related to AA Metabolic Disturbances in Patients With HF

Next, we performed a network analysis to identify gut microbes related to the changes in metabolism of BCAAs and histidine observed in the feces of patients with HF. As shown in Figure 5B, Supplementary Figure 9, the genera *Eubacterium* and *Prevotella* contributed to decreased abundance of microbial genes involved in the biosynthesis of BCAAs in patients with HF. Additionally, the reduction in histidine biosynthesis-related genes was principally attributed to the depletion of *Eubacterium* in patients with HF (Supplementary Figure 10). Furthermore, the abundance of genes involved in histidine degradation in the genus *Bifidobacterium* was higher in patients with HF than in control subjects (Supplementary Figure 10).

## Discussion

Using metagenomic analysis, we demonstrated that the gut microbiota is altered in patients with stable HF. Furthermore, we found that these patients had lower plasma EAA levels than controls. The results of our study provide novel insights into the role of the gut microbiota in AA metabolism among patients with HF. First, we found that gut microbial genes involved in the biosynthesis and degradation of several EAAs were decreased and increased, respectively in patients with HF. Second, the abundance of microbial genes involved in the biosynthesis of BCAAs demonstrated a significant positive correlation with plasma BCAA levels in HF patients, but not in controls. We also found that specific gut microbes, such as the genera *Eubacterium* and *Prevotella*, were associated with decreased abundance of genes involved in the synthesis of microbial EAAs, including BCAAs and histidine.

Previous studies have shown that patients with chronic HF had low total circulating EAA levels associated with severity of the disease (6, 31). Cheng et al. (31) have also reported that a low plasma concentration of total EAA was associated with death or readmission in patients with HF. Therefore, correcting systemic EAA metabolic abnormalities could improve outcomes in patients with HF.

BCAAs, the most abundant subgroup of EAAs, regulate cellular functions through several mechanisms, including the mammalian target of rapamycin (mTOR) pathway (32). BCAA catabolic defects in failing mouse hearts induced by pressure overload or myocardial infarction (MI) cause the accumulation of BCAAs and branched-chain α-keto acids in the heart. This results in the exacerbation of cardiac dysfunction and remodeling via activation of mTOR signaling and promotion of superoxide production (33, 34). In addition, a previous study demonstrated the association of higher circulating BCAA concentrations on admission with an increased risk of future adverse cardiovascular events in patients with ST-segment elevation MI and acute HF (35). In contrast, several studies have reported that BCAAs exhibit beneficial effects against HF. In HF rat models with cardiac cachexia, BCAAs preserved cardiac function and prolonged survival (36). Aquilani et al. (37) demonstrated that oral EAA supplements containing BCAAs improved exercise and walking capacities and decreased plasma lactate and pyruvate levels in patients with sarcopenia and chronic HF. In a preliminary study, oral BCAA supplementation increased serum albumin and decreased cardiothoracic ratio in patients with HF and hypoalbuminemia (38). Furthermore, Fischer's ratio in systolic heart failure (39) and BCAA/total AA ratio in non-ischemic dilated cardiomyopathy (40) have been shown to be positively correlated with left ventricular ejection fraction. Furthermore, low BCAA/total AA ratio was found to be a predictor of composite cardiac events such as hospitalization due to worsening heart failure and cardiac death (40). Since BCAAs promote albumin synthesis (41), BCAAs could be beneficial in patients with HF and malnutrition, sarcopenia, or cachexia. In our study, all the HF patients and controls demonstrated more than 90% intake of the same hospital diet, and liver functions were similar between the two groups. However, plasma albumin levels and Fischer's ratios were lower in patients with HF than in control subjects. It is notable that there were positive correlations between the abundances of microbial genes involved in BCAA biosynthesis and plasma BCAA levels in patients with HF. Moreover, the genera *Eubacterium* and *Prevotella*, which harbor genes involved in several BCAA biosynthesis pathways, were depleted in patients with HF. Collectively, our findings suggest that specific gut microbes may be therapeutic targets for improving systemic BCAA metabolic disturbances in patients with HF.

Histidine exerts antioxidant properties as a scavenger of the hydroxyl radicals and singlet oxygen (42). The enzyme histidine decarboxylase in the heart can convert histidine into histamine, (43) which provokes vasodilatation and positive inotropic effects (44, 45). Intriguingly, low plasma concentrations of histidine were associated with inflammation, oxidative stress, and greater mortality with patients with chronic kidney disease (46) and increased risk of stroke (47). Further, histidine supplementation improved insulin resistance as well as suppressed inflammation and oxidative stress in obese women with the metabolic syndrome (48). In the present study, we found that the abundance of gut microbial genes involved in histidine biosynthesis demonstrated a modest positive correlation with plasma histidine levels in patients with HF. In addition, modest inverse correlations were observed in microbial genes involved in the degradation and transport of histidine with plasma histidine levels in patients with HF. Some discrepancies between the results of BCAAs and histidine in the correlation analysis may be attributed to the unique nature of histidine. This amino acid is referred to as a semi-EAA since by adulthood, humans are capable of producing certain amounts of it. The heart has been reported to secrete most EAAs via active proteolysis and histidine was the most highly secreted EAA (49). As in the case of BCAA biosynthesis, the network analysis revealed that the genus *Eubacterium* substantially contributed to the decreased abundance of microbial genes involved in histidine biosynthesis among patients with HF.

Alanine, one of non-EAAs, was associated with impaired insulin secretion and an increased risk of incident type 2 diabetes in a population-based large cohort (50). In the present study, HF patients had lower plasma alanine levels compared with controls, whereas there were no differences in gut microbial genes involved in the biosynthesis or degradation of alanine between groups.

Several limitations in our study warrant discussion. First, although this cross-sectional study with a small sample size supports the association between gut dysbiosis and the pathophysiology of HF, it does not provide proof of a causal link. Thus, the findings and generalizability of our study need to be verified in future longitudinal clinical studies with larger sample sizes. Interventional clinical or animal studies may be useful in elucidating the role of gut microbiota in AA metabolism among patients with HF. Additionally, the small sample size of this study impeded the determination of factors (i.e., etiological causes and the severity of HF, inflammation, or gut microbiome) as independent predictors of circulating EAA levels. Alterations in gut microbiota in HF patients were also confounded by medication use. While the patients with HF and control subjects were given the same hospital diets at the time of collection of blood and fecal samples, we cannot exclude the possibility that diet before admission affected the gut microbial composition and functions. Lastly, there were no differences in fecal EAA levels between HF patients and control subjects, despite the alterations in microbial genes involved in EAA metabolism. Unfortunately, we did not assess AA absorptive function in the intestine, which may be useful to refer to the point.

## Conclusion

Through whole-genome shotgun sequencing of fecal samples and mass spectrometry-based profiling of AAs, we found that patients with HF demonstrated lower plasma EAA levels compared to controls. Furthermore, we identified a positive correlation between microbial BCAA biosynthesis genes and plasma BCAA levels. Additionally, the observed decreased abundance of microbial genes involved in EAA biosynthesis in the patients with HF was attributed to depletion of *Eubacterium* and *Prevotella*. To the best of our knowledge, the present study is the first to link gut microbiota to AA metabolic disturbances in patients with HF. A recent study demonstrated that individualized nutritional support reduced the risk of major cardiovascular events and mortality in patients with chronic HF (51). Thus, the gut microbiota may be useful in patient nutritional risk stratification. Furthermore, we identified possible therapeutic targets in the gut microbiota for patients with HF.

## Data Availability Statement

The datasets presented in this study can be found in online repositories. The names of the repository/repositories and accession number(s) can be found below: DDBJ Sequence Read Archive, DRA007710.

## Ethics Statement

The study was approved by the Ethics Committee of Kobe University (approval no. 160072 and 170131). The patients/participants provided their written informed consent to participate in this study.

## Author Contributions

ToH conceived and designed the study, recruited patients, analyzed and interpreted the data, as well as wrote and revised the manuscript. TYamas revised the manuscript and provided guidance for the study. TTak, HW, and TYamad analyzed the metagenomic data. TTab and MS conducted the LC-MS/MS analysis as well as analyzed and interpreted the data. YG and TeH conducted the metagenome sequencing. KK conducted the CE-TOFMS analysis and analyzed the data. HT and KM oversaw patient recruitment. K-iH provided guidance for the study. All authors reviewed the manuscript.

## Funding

This work was supported by the Japan Society for the Promotion of Science KAKENHI [Grant Numbers: 17K09497 and 20H03676 (K-iH); 19H03653 and 20K21603 (TYamas); 16H06179 (PAGS)], PRIME from the Japan Agency for Medical Research and Development [18069370 (TYamas)], the Japan Circulation Society Translational Research Foundation (K-iH), Hyogo Science and Technology Association (TYamas), Senshin Medical Research Foundation (TYamas), and Human Metabolome Technologies (ToH).

## Conflict of Interest

KK was employed by the company Human Metabolome Technologies. The remaining authors declare that the research was conducted in the absence of any commercial or financial relationships that could be construed as a potential conflict of interest.

## Publisher's Note

All claims expressed in this article are solely those of the authors and do not necessarily represent those of their affiliated organizations, or those of the publisher, the editors and the reviewers. Any product that may be evaluated in this article, or claim that may be made by its manufacturer, is not guaranteed or endorsed by the publisher.
