# Supplementary material for: Uncovering the Role of Gut Microbiota in Amino Acid Metabolic Disturbances in Heart Failure Through Metagenomic Analysis
Source: Front Cardiovasc Med. 2021 Nov 29;8:789325. doi: 10.3389/fcvm.2021.789325 (PMC8667331; doi:10.3389/fcvm.2021.789325)
Supplement: Supplementary file 2 [file Image_2.pdf]

Figure S2

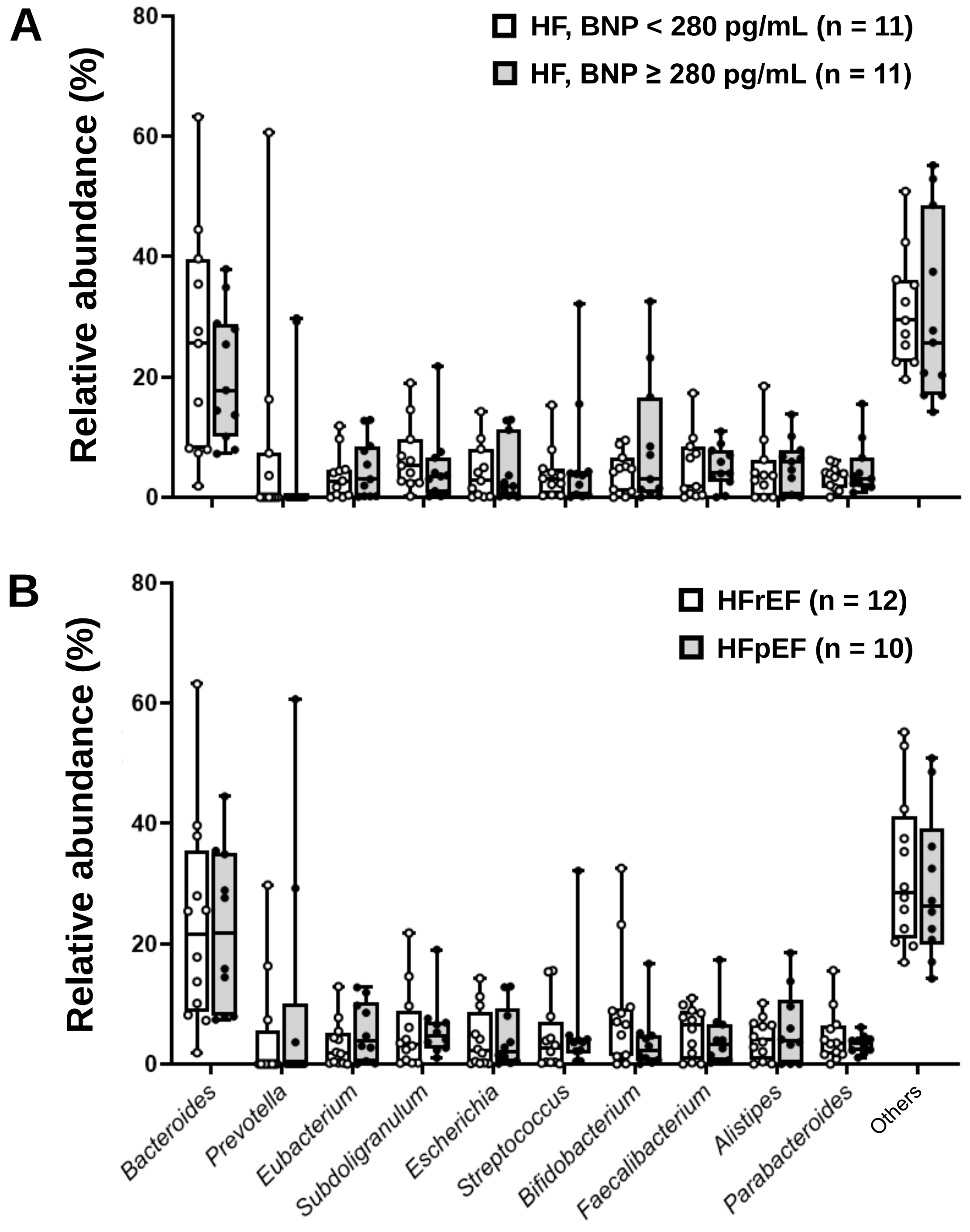

Figure S2. Subgroup analyses of gut microbial compositions based on B-type natriuretic peptide (BNP) levels or left ventricular ejection fraction (LVEF) in heart failure (HF) patients. **(A)** The median level of circulating BNP was 284 pg/mL in HF patients, and we equally divided those patients into 2 groups by a cut-off value of 280 pg/mL. **(B)** HF with reduced ejection fraction (HFrEF) and preserved ejection fraction (HFpEF) were defined as LVEF ≤ 40% and ≥ 50%, respectively. Relative abundances of the top 10 genus-level bacteria in all samples are shown. In the box-and-whisker plot, the middle line represents the median value, the box indicates interquartile range (25th–75th percentiles), and the range bars indicate the maximum and minimum values. Comparisons were carried out using Mann-Whitney U test. There were no differences in relative abundances of each genus between groups.
