# Supplementary material for: Uncovering the Role of Gut Microbiota in Amino Acid Metabolic Disturbances in Heart Failure Through Metagenomic Analysis
Source: Front Cardiovasc Med. 2021 Nov 29;8:789325. doi: 10.3389/fcvm.2021.789325 (PMC8667331; doi:10.3389/fcvm.2021.789325)
Supplement: Supplementary file 3 [file Image_3.pdf]

Figure S3

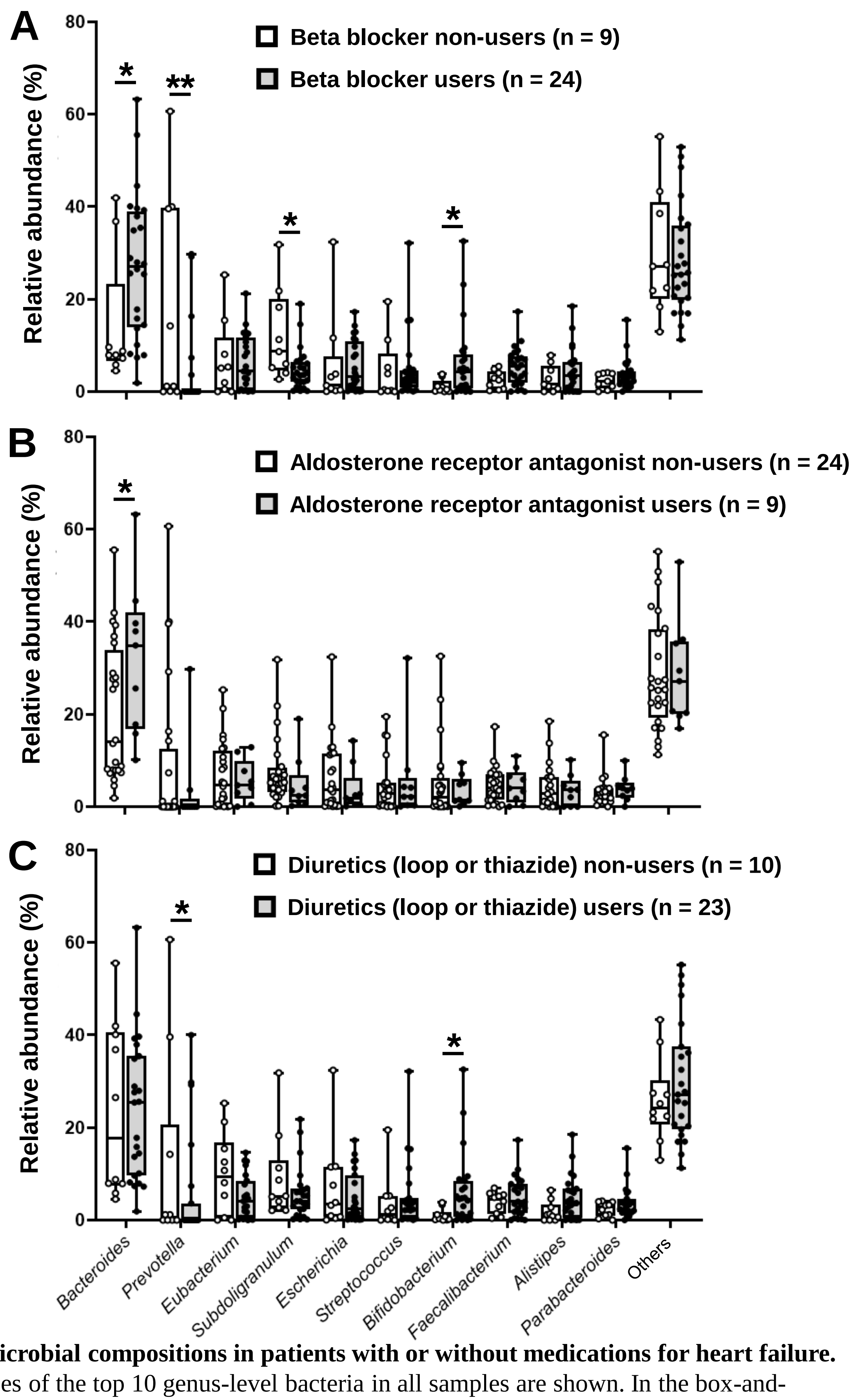

Figure S3. Gut microbial compositions in patients with or without medications for heart failure. Relative abundances of the top 10 genus-level bacteria in all samples are shown. In the box-and-whisker plot, the middle line represents the median value, the box indicates interquartile range (25th–75th percentiles), and the range bars indicate the maximum and minimum values. Comparisons were carried out using Mann-Whitney U test. *P < 0.05, **P < 0.01.
