# Supplementary material for: Uncovering the Role of Gut Microbiota in Amino Acid Metabolic Disturbances in Heart Failure Through Metagenomic Analysis
Source: Front Cardiovasc Med. 2021 Nov 29;8:789325. doi: 10.3389/fcvm.2021.789325 (PMC8667331; doi:10.3389/fcvm.2021.789325)
Supplement: Supplementary file 5 [file Image_5.pdf]

Figure S5

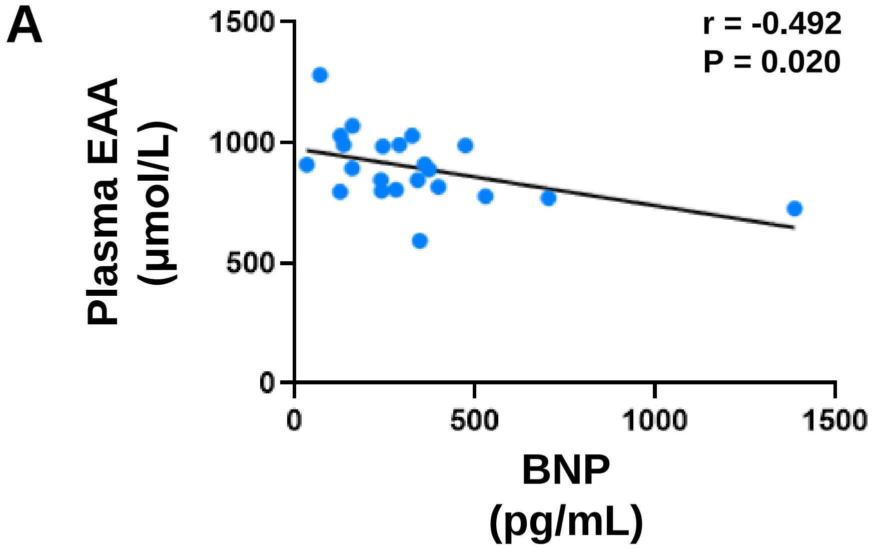

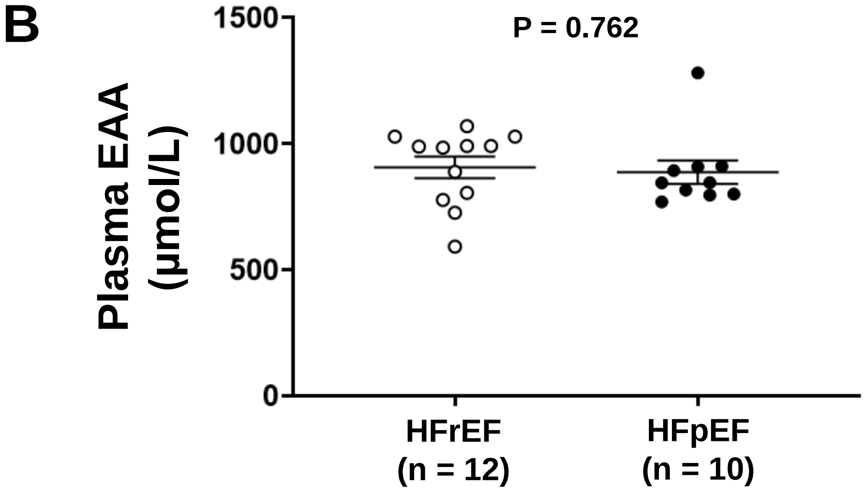

Figure S5. Associations of plasma essential amino acid (EAA) levels with circulating B-type natriuretic peptide (BNP) levels or left ventricular ejection fraction (LVEF).
(A) Relationship between circulating BNP and EAA levels in heart failure (HF) patients (n = 22). Spearman's correlation test was used for the analysis **(B)** Subgroup analysis of plasma EAA levels based on LVEF. HF with reduced ejection fraction (HFrEF) and preserved ejection fraction (HFpEF) were defined as LVEF ≤ 40% and ≥ 50%, respectively. The data are shown as the mean ± SEM. Comparison was carried out using Student's t-test.
