# Supplementary material for: Uncovering the Role of Gut Microbiota in Amino Acid Metabolic Disturbances in Heart Failure Through Metagenomic Analysis
Source: Front Cardiovasc Med. 2021 Nov 29;8:789325. doi: 10.3389/fcvm.2021.789325 (PMC8667331; doi:10.3389/fcvm.2021.789325)
Supplement: Supplementary file 6 [file Image_6.pdf]

Figure S6

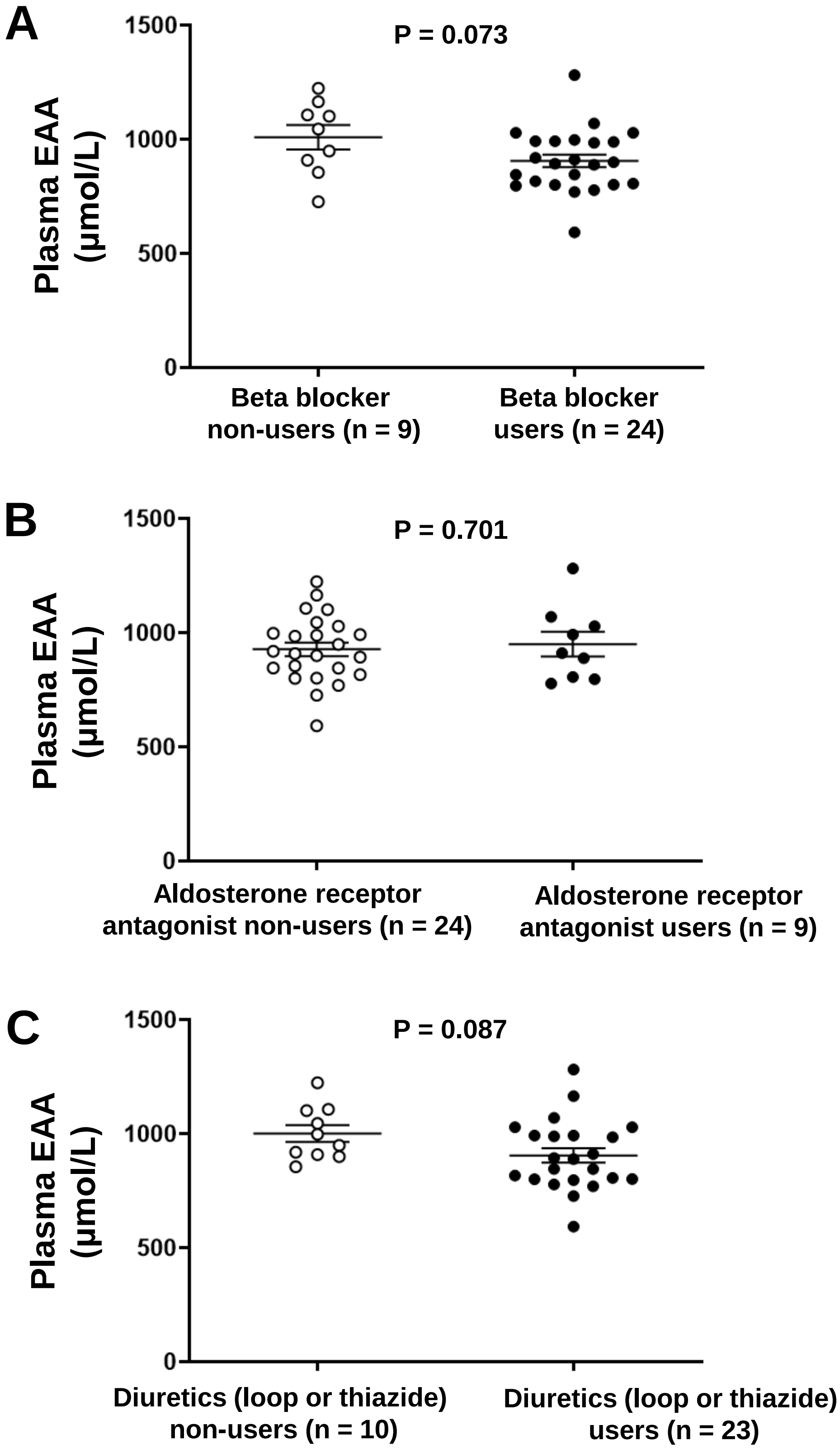

Figure S6. Plasma essential amino acid (EAA) levels in patients with or without medications for heart failure.

The data are shown as the mean ± SEM. Comparisons were carried out using Student's t-test.
