# Supplementary material for: Uncovering the Role of Gut Microbiota in Amino Acid Metabolic Disturbances in Heart Failure Through Metagenomic Analysis
Source: Front Cardiovasc Med. 2021 Nov 29;8:789325. doi: 10.3389/fcvm.2021.789325 (PMC8667331; doi:10.3389/fcvm.2021.789325)
Supplement: Supplementary file 10 [file Image_10.pdf]

Figure S10

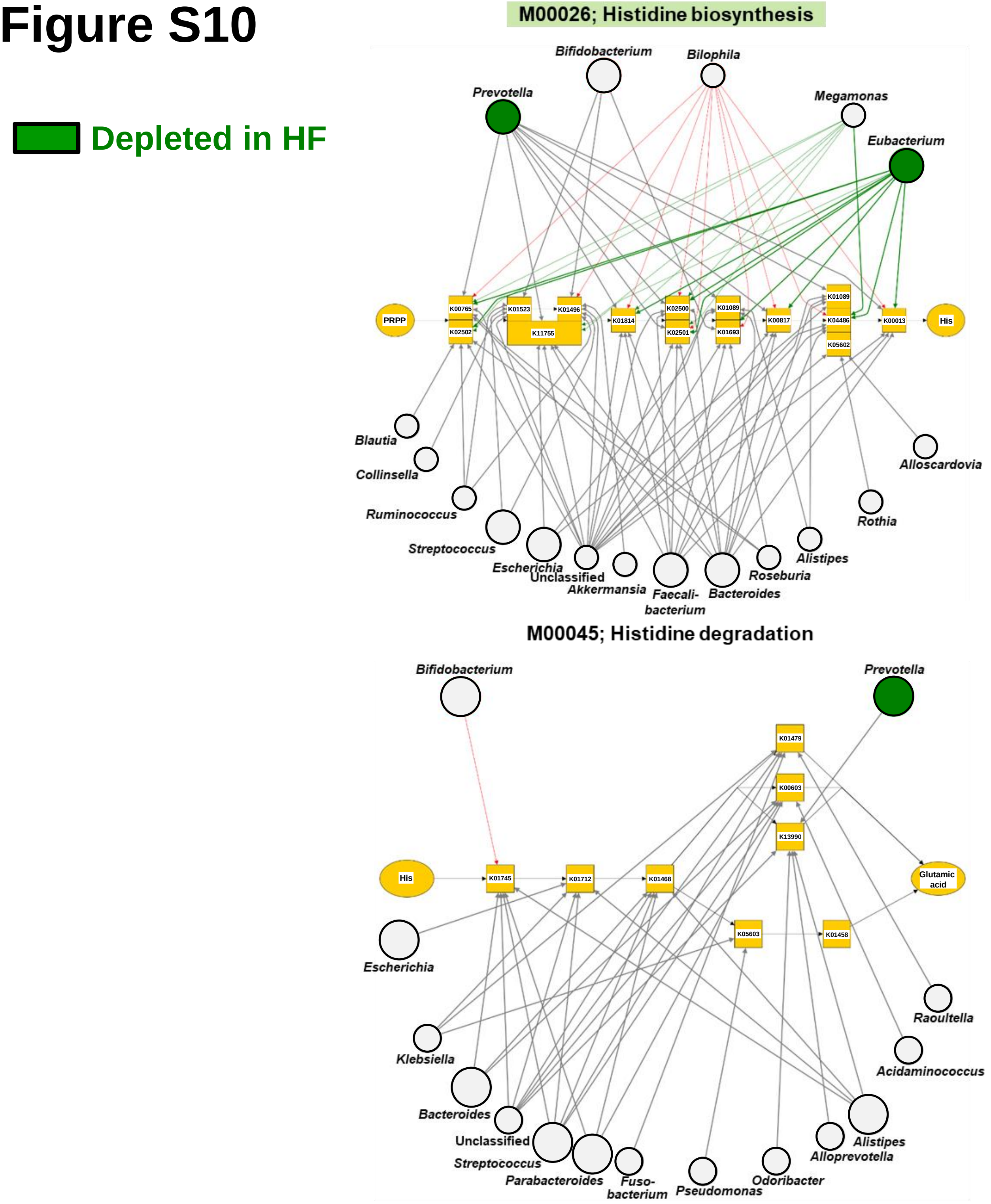

Figure S10. Visualization of genera associated with histidine (His) biosynthesis and degradation. Kyoto Encyclopedia of Genes and Genomes (KEGG) orthology (KO) genes and their associated genera in His biosynthesis (M00026) and degradation (M00045) are shown. The genera depleted in heart failure (HF) patients (n = 22) are indicated by **green circles**. Arrows are drawn from the top 5 abundant genera in each KO gene (**thick arrows**) or from the genera with differentially enriched KO genes between patients with HF (n = 22) and controls (n = 11). **Red arrows** indicate that KO genes possessed by each genus are significantly increased in patients with HF (n = 22). **Green arrows** indicate KO genes significantly decreased in patients with HF (n = 22). **Large circles** indicate the top 10 abundant genera (i.e. *Bacteroides*, *Prevotella*, *Subdoligranulum*, *Eubacterium*, *Escherichia*, *Streptococcus*, *Bifidobacterium*, *Faecalibacterium*, *Alistipes*, and *Parabacteroides*) in all samples (n = 33). PRPP, phosphoribosyl pyrophosphate.
