# Supplementary material for: Uncovering the Role of Gut Microbiota in Amino Acid Metabolic Disturbances in Heart Failure Through Metagenomic Analysis
Source: Front Cardiovasc Med. 2021 Nov 29;8:789325. doi: 10.3389/fcvm.2021.789325 (PMC8667331; doi:10.3389/fcvm.2021.789325)
Supplement: Supplementary file 11 [file Table_1.DOCX]

**Supplementary Table. Analysis of differentially enriched module-based microbial functional pathways between**

**heart failure (HF) patients and control subjects**

| **Module** | **Reporter score** | **Definition** |
| --- | --- | --- |
| M00542 | -6.125 | EHEC/EPEC pathogenicity signature, T3SS and effectors |
| M00023 | -3.800 | Tryptophan biosynthesis, chorismate => tryptophan |
| M00660 | -3.634 | Xanthomonas spp. pathogenicity signature, T3SS and effectors |
| M00127 | -3.529 | Thiamine biosynthesis, prokaryotes, AIR (+ DXP/tyrosine) => TMP/TPP |
| M00157 | -3.101 | F-type ATPase, prokaryotes and chloroplasts |
| M00026 | -3.076 | Histidine biosynthesis, PRPP => histidine |
| M00061 | -3.040 | D-Glucuronate degradation, D-glucuronate => pyruvate + D-glyceraldehyde 3P |
| M00616 | -2.899 | Sulfate-sulfur assimilation |
| M00432 | -2.606 | Leucine biosynthesis, 2-oxoisovalerate => 2-oxoisocaproate |
| M00060 | -2.542 | KDO2-lipid A biosynthesis, Raetz pathway, LpxL-LpxM type |
| M00119 | -2.503 | Pantothenate biosynthesis, valine/L-aspartate => pantothenate |
| M00046 | -2.487 | Pyrimidine degradation, uracil => beta-alanine, thymine => 3-aminoisobutanoate |
| M00845 | -2.472 | Arginine biosynthesis, glutamate => acetylcitrulline => arginine |
| M00899 | -2.403 | Thiamine salvage pathway, HMP/HET => TMP |
| M00866 | -2.389 | KDO2-lipid A biosynthesis, Raetz pathway, non-LpxL-LpxM type |
| M00897 | -2.352 | Thiamine biosynthesis, plants, AIR (+ NAD+) => TMP/thiamine/TPP |
| M00535 | -2.310 | Isoleucine biosynthesis, pyruvate => 2-oxobutanoate |
| M00527 | -2.302 | Lysine biosynthesis, DAP aminotransferase pathway, aspartate => lysine |
| M00122 | -2.209 | Cobalamin biosynthesis, cobyrinate a,c-diamide => cobalamin |
| M00526 | -2.193 | Lysine biosynthesis, DAP dehydrogenase pathway, aspartate => lysine |
| M00117 | -2.130 | Ubiquinone biosynthesis, prokaryotes, chorismate (+ polyprenyl-PP) => ubiquinol |
| M00028 | -2.121 | Ornithine biosynthesis, glutamate => ornithine |
| M00895 | -2.106 | Thiamine biosynthesis, prokaryotes, AIR (+ DXP/glycine) => TMP/TPP |
| M00063 | -2.040 | CMP-KDO biosynthesis |
| M00570 | -2.017 | Isoleucine biosynthesis, threonine => 2-oxobutanoate => isoleucine |
| M00015 | -2.009 | Proline biosynthesis, glutamate => proline |
| M00609 | -1.948 | Cysteine biosynthesis, methionine => cysteine |
| M00573 | -1.940 | Biotin biosynthesis, BioI pathway, long-chain-acyl-ACP => pimeloyl-ACP => biotin |
| M00115 | -1.884 | NAD biosynthesis, aspartate => quinolinate => NAD |
| M00916 | -1.881 | Pyridoxal-P biosynthesis, R5P + glyceraldehyde-3P + glutamine => pyridoxal-P |
| M00010 | -1.876 | Citrate cycle, first carbon oxidation, oxaloacetate => 2-oxoglutarate |
| M00096 | -1.854 | C5 isoprenoid biosynthesis, non-mevalonate pathway |
| M00144 | -1.825 | NADH:quinone oxidoreductase, prokaryotes |
| M00873 | -1.762 | Fatty acid biosynthesis in mitochondria, animals |
| M00913 | -1.738 | Pantothenate biosynthesis, 2-oxoisovalerate/spermine => pantothenate |
| M00874 | -1.734 | Fatty acid biosynthesis in mitochondria, fungi |
| M00935 | -1.718 | Methanofuran biosynthesis |
| M00631 | -1.715 | D-Galacturonate degradation (bacteria), D-galacturonate => pyruvate + D-glyceraldehyde 3P |
| M00123 | -1.678 | Biotin biosynthesis, pimeloyl-ACP/CoA => biotin |
| M00308 | -1.674 | Semi-phosphorylative Entner-Doudoroff pathway, gluconate => glycerate-3P |
| M00019 | -1.651 | Valine/isoleucine biosynthesis, pyruvate => valine / 2-oxobutanoate => isoleucine |
| M00847 | 1.683 | Heme biosynthesis, archaea, siroheme => heme |
| M00169 | 1.692 | CAM (Crassulacean acid metabolism), light |
| M00006 | 1.702 | Pentose phosphate pathway, oxidative phase, glucose 6P => ribulose 5P |
| M00921 | 1.716 | Cyclooctatin biosynthesis, dimethylallyl-PP + isopentenyl-PP => cyclooctatin |
| M00549 | 1.721 | Nucleotide sugar biosynthesis, glucose => UDP-glucose |
| M00377 | 1.742 | Reductive acetyl-CoA pathway (Wood-Ljungdahl pathway) |
| M00053 | 1.743 | Pyrimidine deoxyribonuleotide biosynthesis, CDP/CTP => dCDP/dCTP,dTDP/dTTP |
| M00172 | 1.748 | C4-dicarboxylic acid cycle, NADP - malic enzyme type |
| M00615 | 1.798 | Nitrate assimilation |
| M00095 | 1.802 | C5 isoprenoid biosynthesis, mevalonate pathway |
| M00079 | 1.804 | Keratan sulfate degradation |
| M00741 | 1.870 | Propanoyl-CoA metabolism, propanoyl-CoA => succinyl-CoA |
| M00090 | 1.874 | Phosphatidylcholine (PC) biosynthesis, choline => PC |
| M00151 | 1.973 | Cytochrome bc1 complex respiratory unit |
| M00072 | 1.993 | N-glycosylation by oligosaccharyltransferase |
| M00876 | 2.003 | Staphyloferrin A biosynthesis, L-ornithine => staphyloferrin A |
| M00700 | 2.019 | Multidrug resistance, efflux pump AbcA |
| M00045 | 2.043 | Histidine degradation, histidine => N-formiminoglutamate => glutamate |
| M00725 | 2.063 | Cationic antimicrobial peptide (CAMP) resistance, dltABCD operon |
| M00814 | 2.070 | Acarbose biosynthesis, sedoheptulopyranose-7P => acarbose |
| M00930 | 2.090 | Menaquinone biosynthesis, futalosine pathway |
| M00035 | 2.139 | Methionine degradation |
| M00365 | 2.183 | C10-C20 isoprenoid biosynthesis, archaea |
| M00918 | 2.219 | Aerobactin biosynthesis, lysine => aerobactin |
| M00530 | 2.414 | Dissimilatory nitrate reduction, nitrate => ammonia |
| M00533 | 2.505 | Homoprotocatechuate degradation, homoprotocatechuate => 2-oxohept-3-enedioate |
| M00152 | 2.621 | Cytochrome bc1 complex |
| M00076 | 2.705 | Dermatan sulfate degradation |
| M00077 | 2.705 | Chondroitin sulfate degradation |
| M00563 | 2.813 | Methanogenesis, methylamine/dimethylamine/trimethylamine => methane |
| M00338 | 2.815 | Cysteine biosynthesis, homocysteine + serine => cysteine |
| M00529 | 3.023 | Denitrification, nitrate => nitrogen |
| M00793 | 3.094 | dTDP-L-rhamnose biosynthesis |
| M00860 | 3.098 | Bacillus anthracis pathogenicity signature, polyglutamic acid capsule biosynthesis |
| M00064 | 3.186 | ADP-L-glycero-D-manno-heptose biosynthesis |
| M00307 | 4.005 | Pyruvate oxidation, pyruvate => acetyl-CoA |

The enriched modules in patients with HF (n = 22) and controls (n = 11) are indicated with positive reporter scores and negative

reporter scores, respectively.
